# Sperm quality analyzer: A portable LED array microscope with dark‐field imaging

**DOI:** 10.1002/btm2.10703

**Published:** 2024-08-02

**Authors:** Meng Shao, Changxu Li, Xiaohao Ma, Haoyu Pan, Zeyu Ke, Rui Liu, Zhiguo Zhang, Min‐Cheng Zhong, Yi Wang, Zhensheng Zhong, Fengya Lu, Xunbin Wei, Jinhua Zhou

**Affiliations:** ^1^ School of Biomedical Engineering Anhui Medical University Hefei China; ^2^ Department of Obstetrics and Gynecology, Reproductive Medicine Center First Affiliated Hospital of Anhui Medical University Hefei China; ^3^ Key Laboratory of Measuring Theory and Precision Instrument, School of Instrument Science and Optoelectronics Engineering Hefei University of Technology Hefei China; ^4^ Biomedical Engineering Department and Cancer Hospital and Institute, Key Laboratory of Carcinogenesis and Translational Research Peking University Beijing China; ^5^ Provincial Institute of Translational Medicine Anhui Medical University Hefei China

**Keywords:** dark‐field imaging, LabCASA, LED array microscope, sperm motility

## Abstract

Sperm quality analysis plays an important role in diagnosing infertility, which is widely implemented by computer‐assisted sperm analysis (CASA) of sperm‐swimming imaging from commercial phase‐contrast microscopy. A well‐equipped microscope comes with a high cost, increasing the burden of assessment, and it also occupies a large volume. For point‐of‐care testing (POCT) of sperm quality, these factors are confronted with the challenges of low‐cost and portable instruments. In this study, an encoded light‐emitting diode (LED) array illumination is employed to achieve a portable microscope with multicontrast imaging for sperm quality analysis. This microscopy has dimensions of 16.5 × 14.0 × 25.0 cm, and its dark‐field (DF) imaging provides high‐contrast sperm image data which is suitable for CASA. According to DF imaging, we developed a software of LabCASA, which can used to assess the motility characteristics of sperm. Compared with TrackMate, the difference in motility parameters from our software was less than 10% in the coefficient of variation (CV). The sperm motility parameters vary with the chamber temperature, which further confirms the reliability of our system with DF imaging. The DF imaging provides strong robustness for tracking sperm's motion under different microscopes. For assessment of the motility parameters, our system can work at a lower cost with a plastic structure. This system with DF imaging is suitable for portable POCT of sperm quality analysis, which is highly cost‐effective in resource‐constrained circumstances.

**Translational Impact Statement**

Our designed LED array microscope and developed LabCASA software provide a portable and cost‐effective solution for POCT of sperm motility. The DF imaging provides strong robustness for tracking sperm's motion across imaging platforms. This system allows for sperm quality analysis in resource‐constrained circumstances, contributing to the improvement of the accessibility and affordability of sperm quality analysis.

## 1 INTRODUCTION

Assisted reproductive technology plays a pivotal role in the treatment of infertility, enabling many couples to fulfill their desire for parenthood.^1^, ^2^, ^3^, ^4^, ^5^ The assessment of sperm quality is crucial in this process as it directly impacts the success of fertilization.^6^, ^7^ Currently, methods of analyzing sperm quality primarily rely on microscope‐based examinations and computer‐assisted sperm analysis (CASA) systems,^8^, ^9^, ^10^, ^11^ which are described in the World Health Organization (WHO) Laboratory Manual for the Examination and Processing of Human Semen.^12^ CASA allows us to rapidly assess the parameters, such as sperm concentration, count, and motility. In CASA, high‐contrast imaging for tracking sperms is provided by phase‐contrast microscopy.^8^, ^13^ These parameters provide critical data for disease diagnosis, treatment selection, and research. Although CASA provides the advantages of quantitative analysis and quick assessment, its high cost of phase‐contrast microscope compels many researchers to assess sperm motility using manual or semi‐manual methods.

For male infertility, a low‐cost microscope can be applied to analyze semen, which offers a simple solution to quickly screen. Combined with a smartphone, a single‐ball lens microscope can be convenient at home or on‐site for fertility testing.^14^ Due to obvious spherical aberration, the peripheral view was blurred when the central zone was clear. The foldscope microscope was assembled from folded paper, making it highly flexible, portable, and lightweight.^15^ These two methods still cannot provide the motility parameters. In bright field (BF) imaging from the conventional microscope, the TrackMate plugin is frequently used for particle tracking,^16^ which can quantitatively analyze sperm motility parameters^17^, ^18^ As the imaging contrast is severely affected by out‐of‐focus states for particles, the data process of tracking sperms in BF imaging could be varied and complicated for TrackMate. Therefore, low‐cost and strong robustness methods for sperm quality assessment are crucial in infertility for portable point‐of‐care testing (POCT).

The encoded light‐emitting diode (LED) array is a cost‐effective strategy for microscopy, which can form versatile imaging modes. LED array‐based microscopes can adjust aperture size by LED pattern. Since the illumination patterns of LED array can be controlled through time‐sharing, this approach can provide high‐contrast imaging and computational imaging.^19^, ^20^, ^21^, ^22^, ^23^ After acquiring images through sequential LED illumination, these images including BF and dark field (DF) could be restructured as quantitative phase imaging.^24^, ^25^ Furthermore, Rheinberg illumination can be created by configuring the LEDs to enhance image contrast.^26^, ^27^ Therefore, the LED array‐based microscope could offer a cost‐effective solution for sperm quality analysis with high‐contrast imaging.

Here, we presented a portable sperm quality analyzer, which includes a miniature microscope with a supporting software system of CASA. Utilized an encoded LED array illumination, this microscope can provide multi‐contrast imaging, including BF, DF, and Rheinberg imaging. Furthermore, we developed LabCASA software, which can be used to track sperm trajectories under DF imaging. Compared with the TrackMate plugin, the accuracy of our software has been validated for POCT of sperm motility characteristics. To verify the robustness of the system, swimming sperms were tracked in the DF imaging under different microscopes. The results showed no significant differences in motility. Consequently, this system can be conveniently applied to assess sperm quality with low‐cost and strong robustness, which will be beneficial to sperm‐related studies even in resource‐constrained circumstances.

## 2 MATERIALS AND METHODS

### 2.1 Designing and implementation of the microscope

**FIGURE 1:**
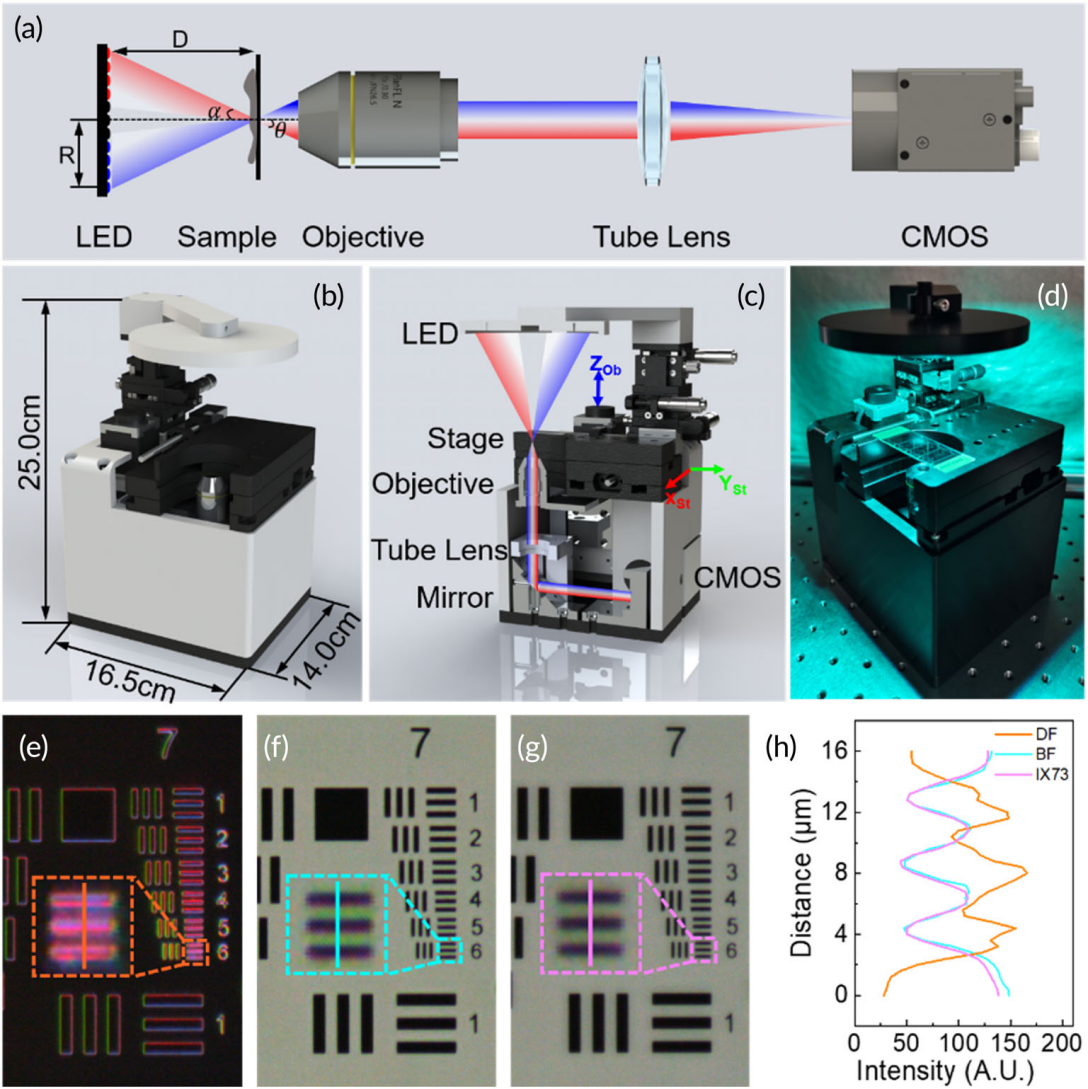
Design and assembly of the light‐emitting diode (LED) array‐based microscope. (a) Schematic of imaging based on encoded LED illumination. (b) Illustration of the device structure in three dimensions. (c) A cross‐sectional diagram of the internal structure. (d) Photograph of the actual device. (e and f) Dark‐field (DF) and bright‐field (BF) images from metal structured microscope (MSM). (g) The BF image from the microscope of Olympus IX73. (h) The plot profiles from the insets.

The schematic representation of the LED array microscope is depicted in Figure 1a. An incoherent LED array source is positioned in the Fourier plane of the optical system. The LED array consists of 61 red‐green‐blue (RGB) LEDs, which are located in five concentric circles with counts of 1, 8, 12, 16, and 24 in sequential order. Each LED within the light source array can be independently controlled, enabling precise illumination of the specimen at specific angles. Therefore, a selected group of LEDs collectively defined the illumination angles.^21^, ^28^ When a specific group of LEDs is illuminated within the numerical aperture of the objective (α < θ), the microscope works in BF imaging. Conversely, a set of LEDs outside the numerical aperture of the objective is only active, DF images can be obtained. Furthermore, the combination of different illumination patterns, including variations in position and wavelength, can provide Rheinberg imaging.

The schematic representation of the three‐dimensional (3D) structure of the device can be demonstrated in Figure 1b. The main components of the microscope include an LED array, an objective, an electronically controlled sample displacement platform, and an industrial camera. Our miniature microscope has a compact and portable design with overall dimensions of 16.5 cm in length, 14.0 cm in width, and 25.0 cm in height. In Figure 1c, the internal structure of the device is depicted through a cross‐sectional view. The light from encoded LEDs is illuminated on the sample, which is collected by the infinity‐corrected objective. It is then modulated into a parallel beam, reflected at an angle of 45‐degree, and passed through a tube lens to image by an industrial camera (MV‐SUA231GC‐T, MindVision, Shenzhen, China). The physical construction of the microscope is assembled by metal components, and the metal structured microscope (MSM) is shown in Figure 1d. This system costs approximately $1200. If we use 3D printing with polylactic acid (PLA) material to manufacture the component of the microscope, the cost can be reduced to approximately 50% ($574). The detailed costs are listed in Supporting Information: Table S1.

Although the objective and camera are selected cost‐effectively, the quality of BF imaging is comparable to the image obtained from the microscope of Olympus IX73. Under DF and BF imaging with a 10× objective (numerical aperture (NA) = 0.3), CNOPTEC, Chongqing, China), element 6 in the 7th group of the United States Air Force MIL‐STD‐150A standard of 1951 (USAF) test target is visible, as illustrated in Figure 1e,f. Compared with the BF image in Figure 1g obtained from the Olympus IX73 microscope, the plot profiles from the colored insets can depict the distribution of a sample, as shown in Figure 1h. The resolution of our device is close to the imaging resolution of the commercial microscope of Olympus IX73. This compact design retains the essential imaging comparable to commercial microscopes and provides flexible control, which could be portable in POCT.

### 2.2 Preparation and analysis of sperm samples

**FIGURE 2:**
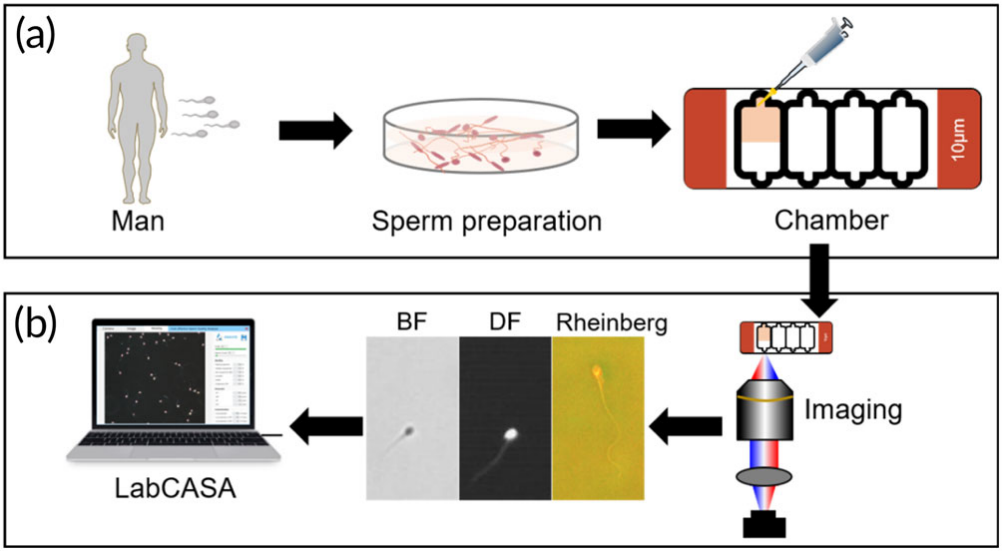
Workflow of analysis of sperm quality. (a) Preparation of sperm sample. Semen was collected from the patient. The nonsperm cells were removed by the sperm swim‐up method. Then diluted sperm solution of 5 μL flowed into the sperm counting chamber. (b) Analysis of sperm motility. The sperms in the chamber were imaged by the encoded LED array microscope with multi‐contrast images. According to dark‐field (DF) imaging, sperm trajectories can be analyzed by LabCASA. BF, bright field.

According to the approval from the Biomedical Ethics Committee of Anhui Medical University (No. 20210744), all the sperm samples used in this study were collected from the Reproductive Medicine Center, the First Affiliated Hospital of Anhui Medical University. To effectively eliminate white blood cells and other nonsperm cells, sperms have been purified from the swim‐up method. The operation steps are depicted in Supporting Information: Section S2. The process of sperm evaluation is outlined in Figure 2. The sperm sample was diluted in a fertilization medium (Cook Medical, United States).^29^ For the diluted sperm, we took 5 μL and introduced it into the sperm counting chamber with a thickness of 10 μm (ML‐CASA10‐4, Song Jing TianLun, Nanning, China). Subsequently, the sample was imaged by the MSM with an encoded LED array. Compared with different LED illumination patterns, DF imaging demonstrated the sperms with high contrast and was employed to track sperms' heads.

In this study, the motility parameters of CASA include curvilinear velocity (VCL), straight‐line velocity (VSL), and average path velocity (VAP). VCL is the average velocity of the actual point‐to‐point path followed by the sperm.^30^ For the *i*th sperm, the velocity can be described as^31^

(1) $${\mathrm{VCL}}_{i}=\frac{\sum_{j=1}^{M}\sqrt{{\left(x_{j+1}-x_{j}\right)}^{2}+{\left(y_{j+1}-y_{j}\right)}^{2}}}{\left(M-1\right)\Delta t},$$

where *x* and *y* are the coordinates of the path, *M* and Δ*t* represent the number of frames and inter‐frame time‐consuming of the video, respectively. VSL refers to the straight‐line distance from the start point to the end point of the sperm. It can be described as follows:

(2) $${\mathrm{VSL}}_{i}=\frac{\sqrt{{\left(x_{M}-x_{1}\right)}^{2}+{\left(y_{M}-y_{1}\right)}^{2}}}{\left(M-1\right)\Delta t},$$

VAP can be calculated from the sperm head along its spatial averaging of the curvilinear trajectory. It can be described as follows:

(3) $${\mathrm{VAP}}_{i}=\frac{\sum_{j=1}^{M-1}\sqrt{{\left({\bar{x}}_{j+1}-{\bar{x}}_{j}\right)}^{2}+{\left({\bar{y}}_{j+1}-{\bar{y}}_{j}\right)}^{2}}}{\left(M-1\right)\Delta t}.$$

In this equation, ${\bar{x}}_{k}=\frac{1}{5}\sum_{i=k-2}^{k+2}x_{i}$ and ${\bar{y}}_{k}=\frac{1}{5}\sum_{i=k-2}^{k+2}y_{i}$. During the testing process, a video is recorded at 30 frames per second (fps) for 1 s to calculate VCL, VSL, and VAP. Additionally, we examined straightness (STR) and linearity (LIN) to assess path curvature. These values were determined by the ratios of VSL/VAP and VSL/VCL, respectively. When we considered the parameter wobble (WOB) to evaluate the side‐to‐side movement of the sperm head, it was determined by the ratio of VAP/VCL.^12^, ^32^ These parameters have been integrated into a software of LabCASA, which is edited by LabVIEW.

### 2.3 Statistical analysis

For the analysis of all sperm variables, the data were first assessed for normality and homoscedasticity using Shapiro–Wilk and Levene tests, respectively. One‐way ANOVA was further used to assess the statistical differences in sperm parameters for the same patient's sample observed in different microscope platforms, for four preparations of the same sample, the differences in parameters were analyzed by one‐way ANOVA. The independent samples *t*‐test was used to assess the differences in sperm parameters obtained from LabCASA and TrackMate. Statistical analysis of parameters was performed using SPSS (version 23.0) software. All data were indicated by means ± standard deviation (SD). The reproducibility of a motion parameter obtained through LabCASA was assessed by the coefficient of variation (CV), which is calculated by dividing the SD of the differences between TrackMate and LabCASA by the overall mean value.

## 3 RESULTS AND DISCUSSION

### 3.1 Multi‐contrast imaging of sperm

**FIGURE 3:**
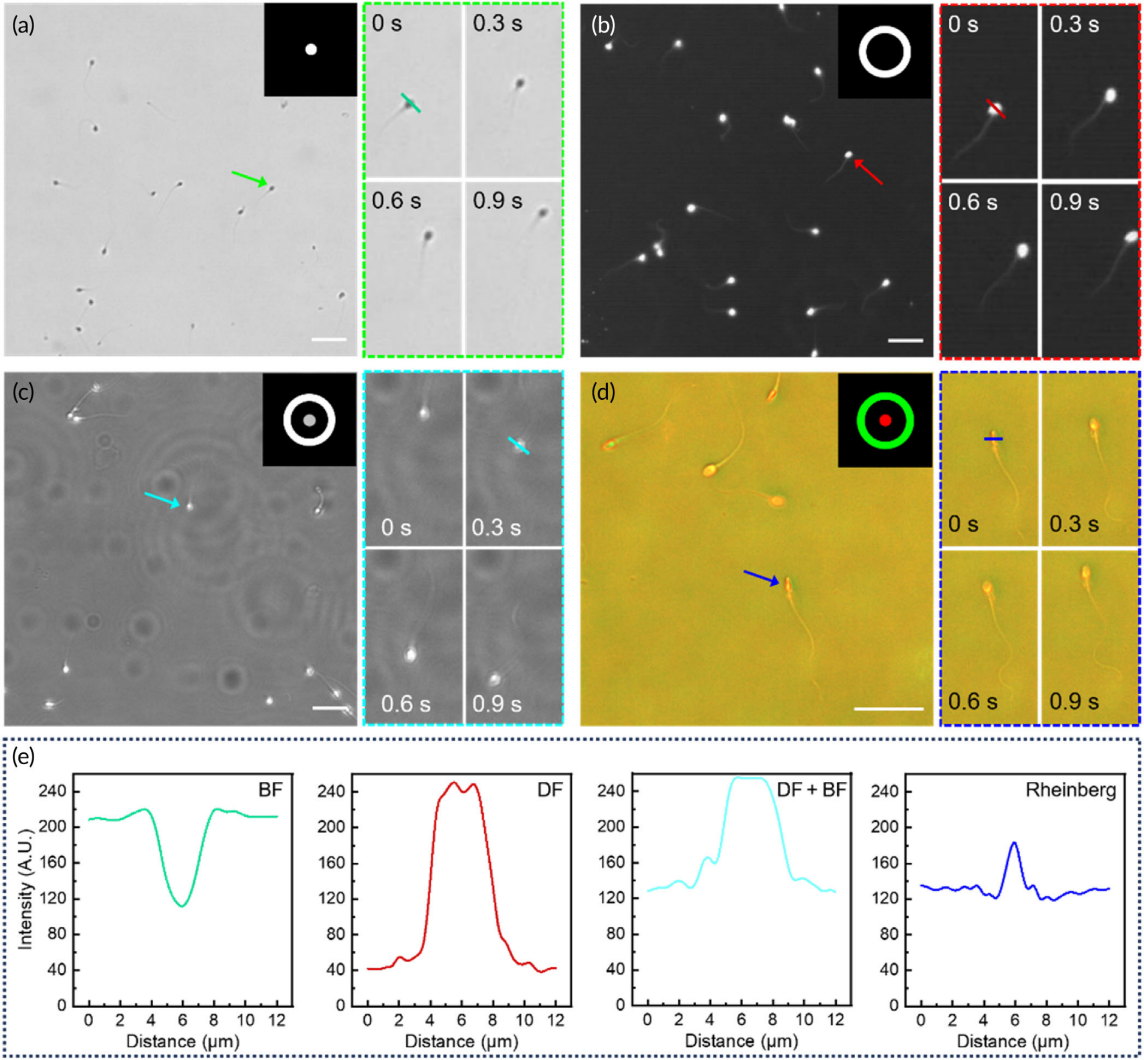
Multicontrast imaging of sperm using our device. (a–c) Images in BF, DF, and the illumination combined bright field (BF) with dark field (DF) under a 10× objective. (d) Rheinberg imaging under a 20× objective. Scale bar: 20 μm. (e) The intensity profiles of a sperm under four different imaging modes.

Different imaging contrast allows for better observation and evaluation of sperm. As depicted in Figure 3, multi‐contrast images of sperm are captured using diverse LED illumination patterns. When a single LED in the central area of the LED array is illuminated, BF images of sperm are obtained, as shown in Figure 3a and Video S1. In such conditions, the sperm morphology is visible against a bright background. However, there may be challenges in determining the threshold for extracting sperm targets due to the relatively lower contrast. This can lead to concerns related to over‐segmentation or under‐segmentation during image processing. When the third ring of LEDs is activated, the acquired images provide a brighter sperm morphology with a darker background, as shown in Figure 3b and Video S2. In this scenario, the contrast of the image is enhanced obviously, which enables a more precise extraction of sperm target regions. Illuminating by both the central LED and the third ring of LEDs simultaneously, as shown in Figure 3c and Video S3, this configuration allows the superposition of the BF imaging with critical illumination and DF imaging. The sperm heads demonstrate bright distribution in a relatively dark background. However, the background is affected by overlay particles out‐of‐plane from central LED illumination. In this imaging mode, the local contrast is high, but it is difficult to segment sperm heads with a uniform threshold.

In our system, the other magnification of the objective also can work with multicontrast imaging. Under the 20× objective, Rheinberg imaging was implemented by setting the LED's color (Figure 3d and Video S4), providing a clear view of the movement of sperm heads and tails. Rheinberg imaging can be considered a specialized variant of DF imaging that allows for optical staining and enhances the contrast of the sample with colors.^33^ This imaging mode is a potential application in reconstructing flagellar wave motion, which will reveal the kinematics of sperm motility.^34^, ^35^, ^36^

In our system, the sperms can be conveniently observed by previous multi‐contrast imaging through illumination patterns. Compared with the four imaging modes, the DF imaging has the best contrast, as shown in Figure 3e. For sperm morphology, Rheinberg imaging provides subcellular structure. However, for tracking sperm motion, high contrast is vital for maintaining a uniform threshold, which is important for traditional segmentation methods. Thus, the subsequent assay of sperm motility is based on DF imaging.

### 3.2 Analysis of motility characteristics of sperm

**FIGURE 4:**
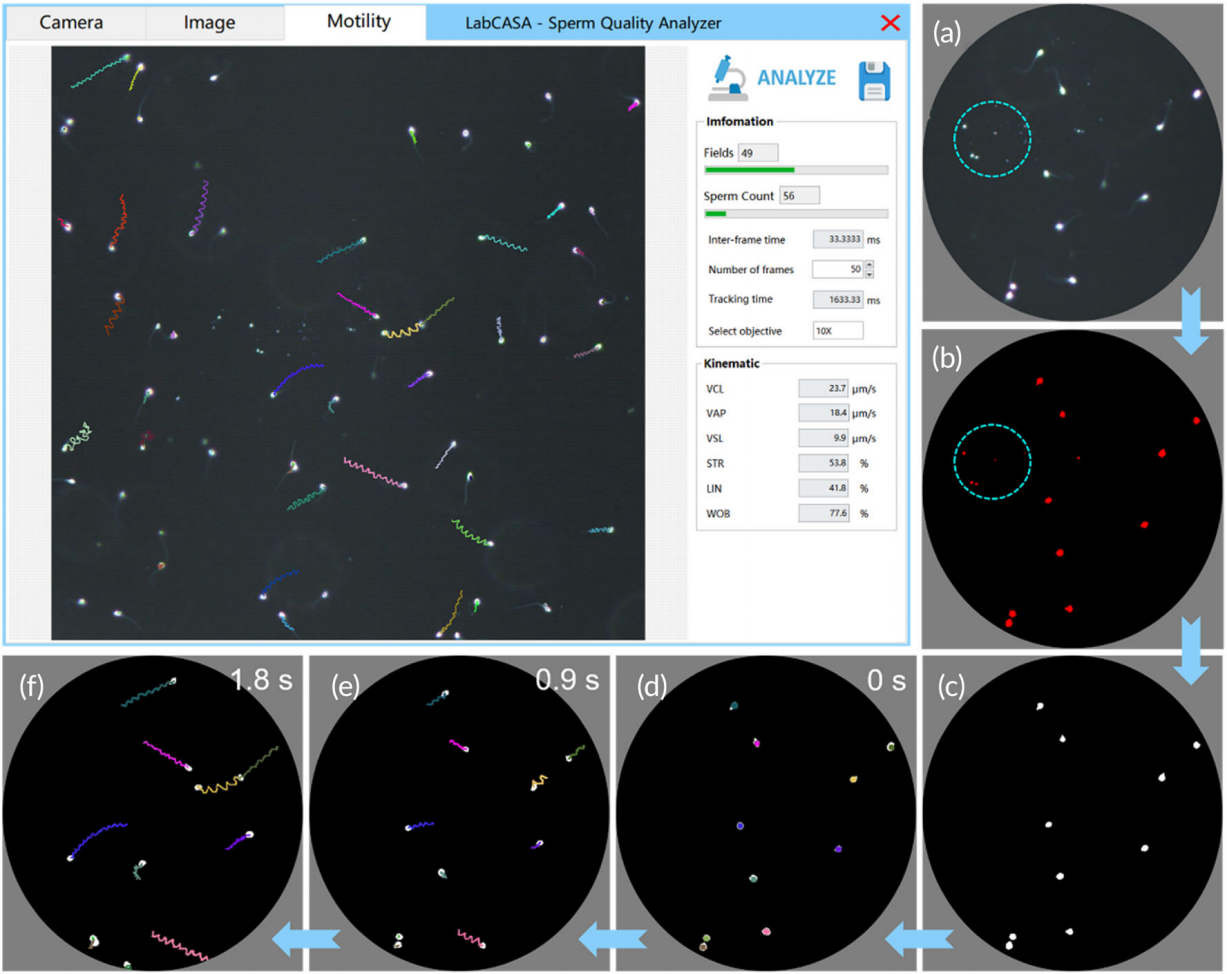
LabCASA for sperm motility parameter analysis. (a) The raw DF image. (b) Threshold segmentation of sperms. (c) Particle filtering algorithm to remove background noise and extract sperm targets. (d)‐(f) sperm targets are recognized, labeled, and tracked.

Sperm motility parameters such as VCL, VSL, and VAP are widely used to evaluate sperm quality.^37^, ^38^, ^39^, ^40^ In scenarios where sperm analysis equipment requires portability and POCT, a LabVIEW‐based analysis software for sperm motility parameters is developed under DF imaging, which is named LabCASA. The software interface is demonstrated in the upper left section of Figure 4. This software comprises three main modules: image acquisition, image processing, and sperm tracking.

The sperm motility parameter can be analyzed by LabCASA. The workflow is illustrated in Figure 4. In the camera module, the sperm motion images can be previewed in real‐time and captured by the camera, as shown in Figure 4a. Subsequently, the captured images undergo threshold segmentation to extract sperm targets in the image module, as shown in Figure 4b. A particle filtering algorithm with an area range of 3–30 μm^2^ is applied to finely extract sperm targets, which effectively eliminates noise from the background. The results of the image processing are delineated by the cyan dashed circles in Figure 4a–c. The detailed steps and parameters are depicted in Supporting Information: Figure S1.

The software proceeds to label the extracted sperm targets with colors, as shown in Figure 4d. Each sperm is represented by a unique color to facilitate tracking. We employed the “Tracking Object” function in LabVIEW to track the sperm. The results are displayed in Figure 4e,f, the sperms' trajectories are demonstrated in Video S5. LabCASA offers the analysis of a range of sperm motility parameters, including VCL, VSL, VAP, STR, LIN, and WOB according to Equations ((1), (2), (3)). This comprehensive approach provides a reliable tool for the holistic evaluation of sperm quality and provides sperm motility parameters with a portable POCT way.

During the analysis of sperm motility characteristics, the image acquisition rate is crucial for CASA analysis. Due to limitations in illumination brightness and camera frame rate, the maximum frame rate for image acquisition is 100 fps. For 100 fps videos, frames are extracted at different intervals using ImageJ, resulting in 10 videos with frame rates ranging from 10 to 100 fps. The sperm motility parameters are then evaluated offline using LabCASA. As shown in Supporting Information: Figure S1, VCL, and VAP stabilize at 100 fps, indicating that this sampling frame rate is sufficient to capture sperm motion details. For POCT, the main factor determining LabCASA's processing speed is computer performance, which limits the video capture frame rate. Supporting Information: Figure S2 illustrates the correlation between the designated frame rate and the actual acquisition frame rate on two different computers. A computer equipped with an i7‐12,700 central processing unit (CPU) achieves roughly 32 fps during real‐time processing. In subsequent experiments, this computer configuration was used to run LabCASA. The sperm image sequence was acquired for 1 s at 30 fps, which is within the 20–60 fps range reported in the literature. If real‐time analysis is not needed, our system can perform offline analysis after capturing at 100 fps for 1 s. We estimate that the total processing time would not exceed 5 seconds, enabling relatively fast analysis of sperm motion parameters.

### 3.3 Comparison of sperm motility between LabCASA and TrackMate

**FIGURE 5:**
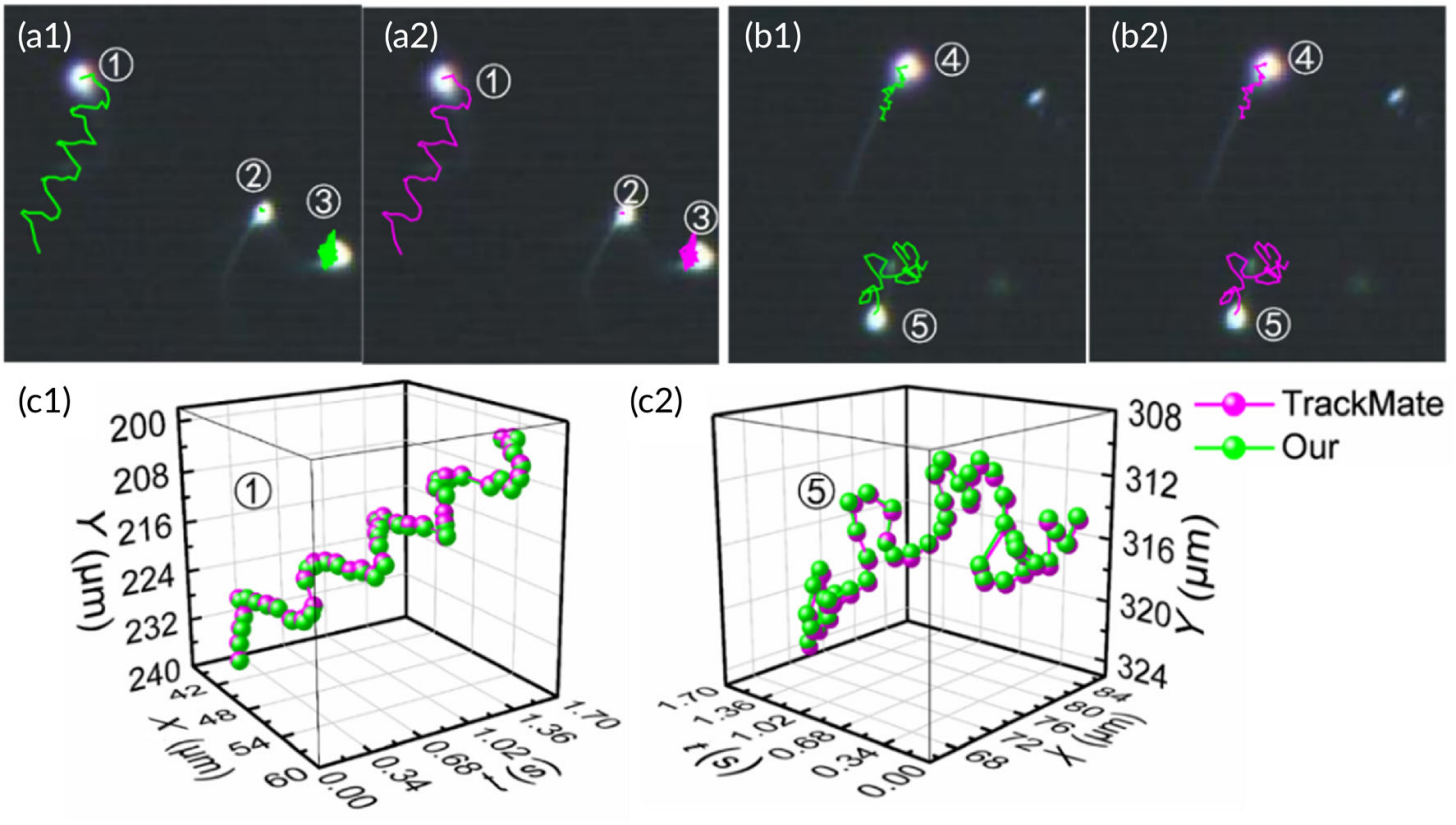
Comparison of the sperm trajectories tracked from LabCASA with TrackMate. The results from LabCASA are shown in (a1) and (b1). The results from TrackMate are shown in (a2) and (b2). The trajectories of sperm labeled as 1 and 5 are shown in (c1) and (c2).

To validate the reliability of our software, the sperm trajectories tracked by the LabCASA software are compared with those tracked by the TrackMate plugin. Consecutive 50 frames of sperm images were processed with LabCASA and TrackMate, which exported the coordinates of each sperm in each frame. The processing steps of TrackMate can be depicted in Supporting Information: Section S4. Then, MATLAB (version 2022; MathWorks) is used to analyze motility parameters, and draw sperm trajectories onto the final frame of the sperm images, as illustrated in Figure 5. The green paths represent sperm trajectories tracked by the LabCASA software, as depicted in Figure 5a1,b1. It is noteworthy that LabCASA not only excels in tracking simple sperm trajectories but also demonstrates remarkable performance in dealing with complex trajectories. In contrast, the tracking results obtained with TrackMate are presented in Figure 5a2,b2. These corresponding trajectories of sperms have similar geometry shapes.

For a more explicit validation, trajectory points in three dimensions of positions and time for the sperms marked as numbers 1 and 5 we plotted in Figure 5c1,c2. The validation results reveal a substantial overlap between the magenta and green paths in terms of position and shape, further substantiating the reliability of LabCASA software in sperm trajectory tracking.

To validate the stability of LabCASA, video data from 10 fields of view were obtained from a single male sample. Subsequently, sperm motion parameters within these fields were analyzed simultaneously using both LabCASA and TrackMate. The statistical results of these motility parameters are presented in Table 1. The parameters detected by LabCASA, including VCL, VSL, and VAP, are consistent with corresponding results detected by TrackMate. Indeed, various CASA systems can significantly impact sperm motility parameters.^9^ Due to the complexity of CASA systems, they are likely to be highly influenced by algorithm settings, such as threshold‐based segmentation algorithms. Another factor that may influence CASA results is the presence of out‐of‐focus sperm that cannot be detected.^41^ The CV was used to measure the reproducibility of LabCASA as a replacement for TrackMate software. For all the analyzed motility parameters, the CV values are less than 4%, which meets the requirement of below 10%.^42^, ^43^ This indicates the suitability and reliability of LabCASA as an alternative to TrackMate software. Furthermore, an independent sample *t*‐test was conducted to evaluate the differences between LabCASA and TrackMate. The test results indicate that all *p*‐values are greater than 0.05, demonstrating no significant difference between the two methods.

**TABLE 1:** Comparison of sperm motility parameters evaluated through LabCASA and TrackMate.

	VCL (μm/s)	VAP (μm/s)	VSL (μm/s)	LIN (%)	STR (%)	WOB (%)
LabCASA	26.7 ± 2.3	13.7 ± 1.9	16.2 ± 1.7	37.3 ± 2.9	62.3 ± 2.8	52.1 ± 1.9
TrackMate	26.1 ± 2.8	13.7 ± 2.1	15.9 ± 2.1	37.4 ± 2.8	64.5 ± 3.3	51.1 ± 2.0
CV (%)	3.27	3.31	3.68	3.39	3.58	2.48
*t*	0.537	0.046	0.336	0.138	1.614	1.174
*p*	0.605	0.655	0.521	0.645	0.253	0.555

Abbreviations: CV, coefficient of variation; LIN, linearity; STR, straightness; VAP, average path velocity; VCL, curvilinear velocity; VSL, straight‐line velocity; WOB, wobble.

To validate the repeatability of the system, four different operations of sperm assay for the same samples have been conducted. According to the WHO laboratory manual for microscope evaluation, each sample selects five fields of view and counts beyond 200 sperms. The evaluation results from the LabCASA system are shown in Table 2. Using one‐way ANOVA, sperm motility parameters have been compared with four different experiments. *F*‐test results indicate *p* > 0.05, indicating no significant difference between the operations. This demonstrates the system has high repeatability and reliability.

**TABLE 2:** The motility parameters in four different experiments detected by LabCASA.

	Number (mean ± SD)				*F*	*p‐*Value
	No. 1	No. 2	No. 3	No. 4		
VCL (μm/s)	29.0 ± 1.2	28.6 ± 0.7	29.5 ± 1.6	28.7 ± 1.5	0.428	0.736
VSL (μm/s)	11.1 ± 1.2	11.7 ± 1.6	10.6 ± 0.5	10.7 ± 1.0	0.884	0.470
VAP (μm/s)	15.2 ± 0.9	15.3 ± 1.3	15.1 ± 0.8	15.0 ± 1.1	0.074	0.973
LIN (%)	30.2 ± 3.2	31.6 ± 4.2	31.3 ± 1.6	31.1 ± 1.7	0.178	0.910
STR (%)	55.4 ± 4.5	56.7 ± 4.2	58.2 ± 3.5	57.0 ± 1.3	0.509	0.682
WOB (%)	47.8 ± 1.3	47.8 ± 2.2	49.7 ± 1.8	48.6 ± 1.4	1.428	0.271

Abbreviations: LIN, linearity; SD, standard deviation; STR, straightness; VAP, average path velocity; VCL, curvilinear velocity; VSL, straight‐line velocity; WOB, wobble.

To validate the feasibility of the system across multiple samples, we have analyzed sperm samples from three patients. In our analysis, the number of sperms is larger than 200. Table 3 presents the sperm parameters of the three patients after processing with LabCASA. To validate the accuracy of LabCASA, the sperm videos of the three patients were also processed using TrackMate. After *t*‐tests, all *p*‐values are greater than 0.05, indicating no significant differences in the two processing methods. This confirms the reliability of LabCASA.

**TABLE 3:** The motility parameters of sperm from different patients.

	Patient 1		Patient 2		Patient 3	
	LabCASA	TrackMate	LabCASA	TrackMate	LabCASA	TrackMate
VCL (μm/s)	28.3 ± 0.5	28.5 ± 1.0	29.4 ± 1.2	30.2 ± 1.8	42.1 ± 1.8	43.2 ± 1.4
VAP (μm/s)	11.0 ± 1.0	11.5 ± 0.9	14.4 ± 0.9	14.9 ± 1.6	22.0 ± 2.4	23.3 ± 2.2
VSL (μm/s)	14.9 ± 0.6	15.2 ± 0.8	17.8 ± 0.8	18.3 ± 1.3	26.0 ± 1.7	27.1 ± 1.6
LIN (%)	30.2 ± 2.2	31.6 ± 2.2	39.9 ± 2.9	40.6 ± 3.0	43.0 ± 2.4	44.9 ± 1.6
STR (%)	55.6 ± 3.4	57.7 ± 3.7	65.6 ± 3.2	67.6 ± 3.1	68.9 ± 1.7	70. 6 ± 1.4
WOB (%)	47.7 ± 1.0	49.2 ± 0.9	55.2 ± 3.1	56.1 ± 2.4	55.9 ± 1.7	57.3 ± 0.9

Abbreviations: LIN, linearity; STR, straightness; VAP, average path velocity; VCL, curvilinear velocity; VSL, straight‐line velocity; WOB, wobble.

### 3.4 Assess the effect of temperature on sperm

**FIGURE 6:**
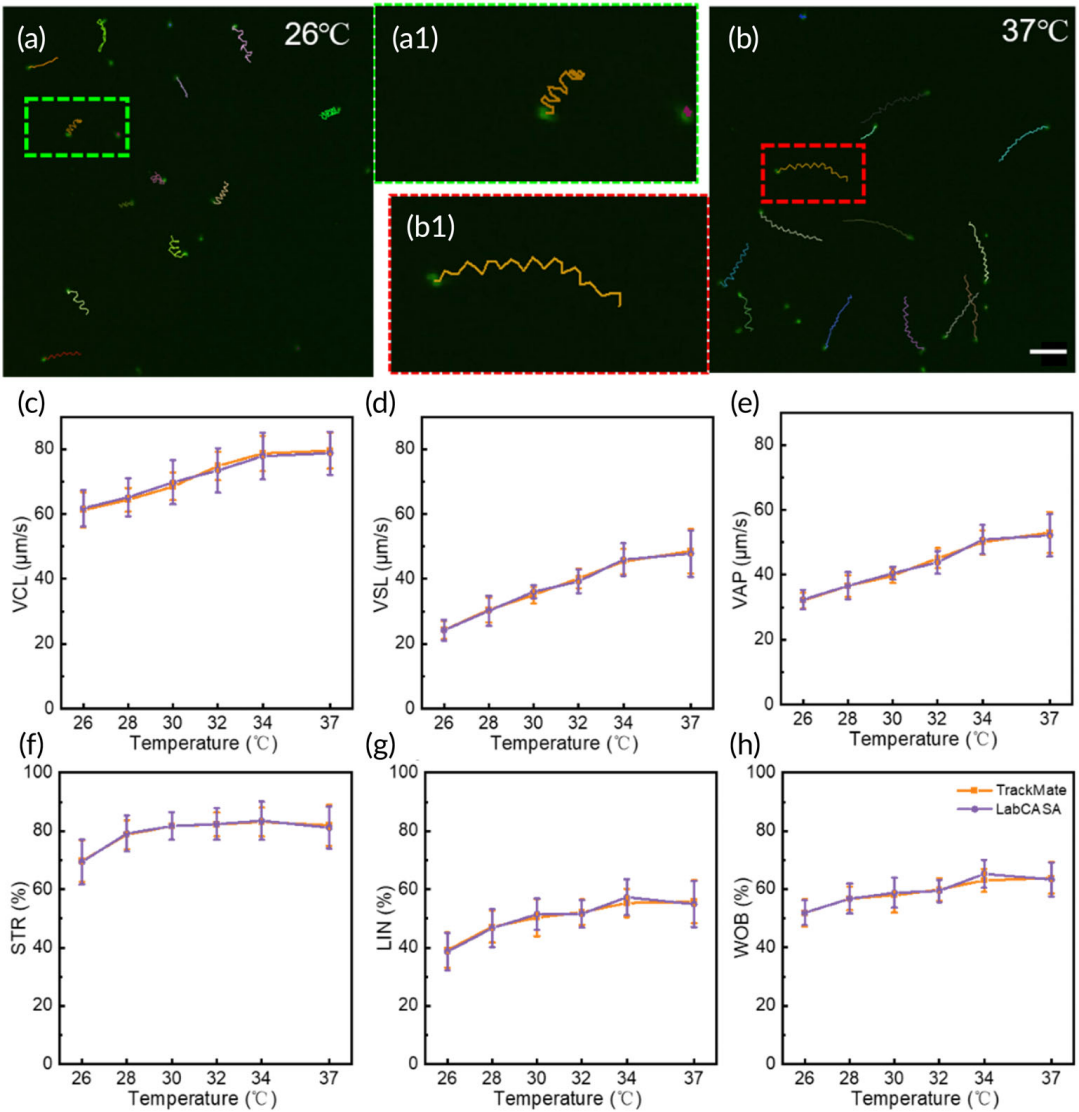
The influence of temperature on sperm motility. (a) and (b) The movement trajectories of sperm under conditions of 26°C and 37°C. Scale bar: 20 μm. (c)–(h) Relationship between six parameters of sperm motility with chamber temperature. LIN, linearity; STR, straightness; VAP, average path velocity; VCL, curvilinear velocity; VSL, straight‐line velocity; WOB, wobble.

Furthermore, LabCASA is utilized to systematically evaluate the impact of temperature on sperm motility, which is a critical factor in optimizing the detection of sperm quality. By subjecting sperm samples to varying temperature conditions within a controlled environment, we aimed to investigate how temperature fluctuations influence the motility characteristics of sperm. The temperature control device has already been thoroughly described in our previous research.^44^ In simple terms, the ITO heater directly contacts the sample chamber, and temperature monitoring and control are achieved through feedback from a temperature‐sensitive sensor. At controlled temperatures of 26 and 37°C, sperm motion trajectories are demonstrated in Figure 6a,b, respectively. It is observed that sperm velocity significantly increases at higher temperatures. However, the amplitude of sperm wobbling does not exhibit significant changes.

The motility parameters varying with temperature were analyzed in Figure 6c–f. The sperm sample chamber was controlled at six temperature gradients 26, 28, 30, 32, 34, and 37°C. At each temperature, 800 sperms from 10 fields of view were tracked by LabCASA and TrackMate, respectively. The parameters from LabCASA are close to the corresponding results from TrackMate. According to VCL, VSL, and VAP, these parameters show an increasing trend in sperm motility, consistent with existing literature.^45^, ^46^, ^47^, ^48^ With the temperature increasing, there is a linear increase in VCL, VAP, VSL, and LIN of the sperm. These parameters reflect the speed and linearity of sperm movement. The observed results may be attributed to the influence of temperature on viscosity. As temperature increases, seminal fluid may become less viscous, allowing sperm to move more easily.^49^ This reduction in fluid viscosity can positively impact parameters related to sperm velocity and linearity. However, STR and WOB do not show significant changes with increasing temperature. This is consistent with the results displayed in the Figure 6a,b. This comprehensive analysis provides valuable insights into the sensitivity of sperm motility to temperature variations.

### 3.5 Robustness of DF imaging for tracking sperms

**FIGURE 7:**
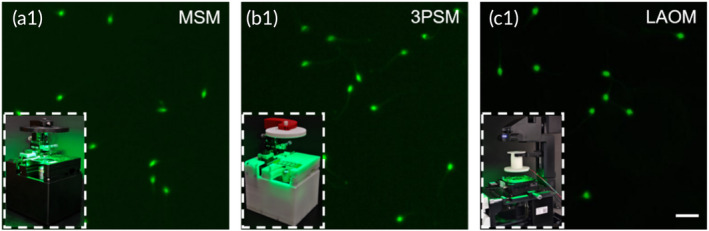
The dark‐field (DF) images from metal structured microscope (MSM), three‐dimensional printing‐structure‐based microscope (3PSM), and LED array‐based Olympus microscope (LAOM). Scale bar: 20 μm.

To validate the robustness of the DF imaging for tracking sperms, the motility parameters were analyzed under three kinds of microscope. We manufactured a 3D‐printing‐structure‐based microscope (3PSM) with PLA material, which had the same structure as the previous MSM. To implement the DF imaging under Olympus IX73, the illumination of the commercial microscope was modified with the same encoded LED array. The LED array‐based Olympus microscope (LAOM) has the same imaging contrast as the other two kinds of microscope. The LAOM can achieve four kinds of imaging contrast. When comparing the four imaging contrasts between LAOM and MSM, sperm imaging quality is very close (see Supporting Information: Figure S2). The DF images of sperm from three kinds of microscope are shown in Figure 7. The quality of sperm imaging is nearly identical in the three DF images. Compared with LAOM and MSM, 3PSM is a plastic‐based microscope that also offers DF imaging, which means this microscope has lower costs for CASA. After tracking sperms beyond the count of 200, the motility parameters tested from the three kinds of microscopes are listed in Table 4. All the videos were recorded from the same sample and processed by LabCASA software. According to one‐way ANOVA analysis, the *p*‐values for all parameters are greater than 0.05. This indicates that there are no significant differences in the corresponding parameters detected by the three kinds of microscopes.

**TABLE 4:** Sperm motility parameters under three kinds of microscope.

	Microscope (mean ± SD)			*F*	*p*‐Value
	3PSM	MSM	LAOM		
VCL (μm/s)	30.1 ± 1.1	29.4 ± 0.9	28.8 ± 0.7	2.368	0.136
VSL (μm/s)	14.1 ± 0.7	14.5 ± 0.8	14.9 ± 0.9	1.254	0.320
VAP (μm/s)	17.4 ± 0.8	17.8 ± 0.8	18.0 ± 0.4	0.916	0.426
LIN (%)	38.4 ± 1.3	39.7 ± 2.8	41.0 ± 1.4	2.213	0.152
STR (%)	67.8 ± 1.9	65.5 ± 3.4	66.8 ± 1.9	1.069	0.374
WOB (%)	52.5 ± 1.4	55.2 ± 3.2	55.7 ± 1.5	2.988	0.089

Abbreviations: 3PSM, three‐dimensional printing‐structure‐based microscope; CV, coefficient of variation; LAOM, LED array‐based Olympus microscope; LIN, linearity; MSM, metal structured microscope; SD, standard deviation; STR, straightness; VAP, average path velocity; VCL, curvilinear velocity; VSL, straight‐line velocity; WOB, wobble.

Since sperm tracking works well under all three kinds of microscopes, DF imaging can be applied to analyze sperm motility across different imaging platforms. For the miniature structure, the MSM has better mechanical stability than the 3PSM. However, the cost of 3PSM is lowest for DF imaging of sperms. Due to limitations in the control program and the use of a low‐cost camera, our system can provide tracking results of sperms in real‐time at 30 fps. If our system works in an offline treatment mode, the acquisition can be up to 100 fps. In this case, the total time for acquisition and treatment would be below 5 s. Our system can not only complete the assessment of the motility parameters within a short time but also work at a low cost. This makes the system suitable for portable POCT of sperm quality analysis.

## 4 CONCLUSIONS

Sperm quality assessment is crucial for diagnosing infertility and guiding reproductive treatments, but the high costs of commercial CASA solutions have limited their widespread adoption, posing a barrier to many laboratories and research groups. This study introduces an approach to solve these challenges by utilizing a miniatured microscope with high‐contrast imaging and tracking software called LabCASA. This system can obtain good imaging quality with a compact structure measuring 16.5 × 14.0 × 25.0 cm and cost‐effective components. Additionally, we have developed and rigorously validated LabCASA software for motility assessment in DF imaging, which exhibits strong robustness for tracking sperms across imaging platforms. This approach could be applied to portable POCT of sperm motility. In resource‐constrained circumstances, the components of this microscope can be manufactured by 3D printing with PLA material. This way will significantly reduce the economic burden associated with sperm quality analysis.

## AUTHOR CONTRIBUTIONS

**Meng Shao:** Data curation; investigation; methodology; writing – original draft; writing – review and editing. **Changxu Li:** Data curation; investigation; methodology; writing – review and editing. **Xiaohao Ma:** Investigation. **Haoyu Pan:** Investigation. **Zeyu Ke:** Investigation. **Rui Liu:** Investigation. **Zhiguo Zhang:** Investigation; methodology. **Min‐Cheng Zhong:** Investigation. **Yi Wang:** Investigation. **Fengya Lu:** Investigation. **Zhensheng Zhong:** Investigation. **Xunbin Wei:** Investigation; methodology. **Jinhua Zhou:** Conceptualization; funding acquisition; methodology; supervision; writing – original draft; writing – review and editing.

## CONFLICT OF INTEREST STATEMENT

The authors declare no conflicts of interest.

## Supporting information


**Data S1.** Supporting Information.

**Video S1.** The magnification of an object lens is generally expressed as 10×, 20A×. BF imaging of sperm under a ×10 objective.

**Video S2.** DF imaging of sperm under a 10 objective.

**Video S3.** Combined BF with DF imaging of sperm under a 10 objective.

**Video S4.** Rheinberg imaging of sperm under a 20 objective.

**Video S5.** LabCASA for sperm motility parameters analysis.

## Data Availability

Data underlying the results presented in this article are not publicly available at this time but may be obtained from the authors upon reasonable request.
