# Supplementary material for: Sperm quality analyzer: A portable LED array microscope with dark‐field imaging
Source: Bioeng Transl Med. 2024 Aug 2;9(6):e10703. doi: 10.1002/btm2.10703 (PMC11558194; doi:10.1002/btm2.10703)
Supplement: Supplementary file 1 — Data S1. Supporting Information. [file BTM2-9-e10703-s002.docx]

**Supplementary Information**

**Sperm Quality Analyzer: A Portable LED Array Microscope with Dark-field imaging**

Meng Shao^1^, Changxu Li^1^, Xiaohao Ma^1^, Haoyu Pan^1^, Zeyu Ke^1^, Rui Liu^1^, Zhiguo Zhang^2^, Min-Cheng Zhong^3^, Yi Wang^1^, Zhensheng Zhong ^1^, Fengya Lu^1^, Xunbin Wei^1, 4^, Jinhua Zhou^1, 5^

1. School of Biomedical Engineering, Anhui Medical University, Hefei, 230032, China
2. Department of Obstetrics and Gynecology, Reproductive Medicine Center, the First Affiliated Hospital of Anhui Medical University, Hefei, 230022, China
3. Key Laboratory of Measuring Theory and Precision Instrument, School of Instrument Science and Optoelectronics Engineering, Hefei University of Technology, Hefei 230009, China
4. Biomedical Engineering Department and Cancer Hospital and Institute, Key Laboratory of Carcinogenesis and Translational Research, Peking University, 100081, Beijing, China
5. Anhui Provincial Institute of Translational Medicine, Anhui Medical University, Hefei 230032, China

Meng Shao and Changxu Li are contributed equally to this work.

**Corresponding Author:**

Zhiguo Zhang, Department of Obstetrics and Gynecology, Reproductive Medicine Center, the First Affiliated Hospital of Anhui Medical University, Hefei, 230022, China

Email: [zzg_100@163.com](mailto:zzg_100@163.com)

Xunbin Wei, School of Biomedical Engineering, Anhui Medical University, Hefei, 230032, China

Email: xwei@bjmu.edu.cn

Jinhua Zhou, School of Biomedical Engineering, Anhui Medical University, Hefei, 230032, China;

Email: zhoujinhua@ahmu.edu.c[n](mailto:zhongmch@hfut.edu.cn)

**VIDEOS**

**Visualization 1.** BF imaging of sperm under a 10X objective.

**Visualization 2.** DF imaging of sperm under a 10X objective.

**Visualization 3.** Combined BF with DF imaging of sperm under a 10X objective.

**Visualization 4.** Rheinberg imaging of sperm under a 20X objective.

**Visualization 5.** LabCASA for sperm motility parameters analysis.

**S1** **The** **cost of a miniature microscope**

**Table S1** provides the approximate costs of the major components. With metal fabrication, the system costs about $1210. However, using PLA-based 3D printing, the cost is reduced by approximately 50% to around $574.

**Table S1** Approximate cost of main components

| **SL.** | **Component name** | **Manufacturer and model number** | **Price**  **(USD)** |
| --- | --- | --- | --- |
| 1 | LED array | WS2812, Worldsemi, China | 8 |
| 2 | LED positioning two-dimensional adjustment bracket | LX40, Hengyang Guangxue, China | 38 |
| 3 | Controllers - Arduino | Arduino Nano | 22 |
| 4 | Camera | MV-SUA231GC-T, MindVision, China | 350 |
| 5 | Tube lens | GCL-010207, *ϕ=*25.4, *f=*175.0, Daheng Optics, China | 45 |
| 6 | Objective | 10X, Infinity, CNOPTEC, China | 57 |
| 7 | Metal structure fabrication |  | 690 |
|  |  | **Total cost of metal structure adoption** | **1210** |
| 7 | PLA-based 3D printed structures |  | 42 |
|  |  | **Total cost of adopting PLA-based 3D printed structures** | **574** |

**S2** **Operation steps for preparing sperm samples**

Semen pretreatment can effectively eliminate white blood cells and other non-sperm cells. The processing method follows the swim-up method described in the fifth edition of the WHO Laboratory Manual for the Examination and Processing of Human Semen. The steps are as follows:

1. Place 1 mL of semen into a 15 mL sterile conical centrifuge tube.
2. Slowly add 1.2 mL of culture medium.
3. Tilt the centrifuge tube at 45° to increase the contact area, and incubate at 37 ℃ for 1 hour.
4. Place the centrifuge tube vertically and aspirate 1 mL at the top of the culture medium containing highly active sperm.
5. Add 2 mL of diluted culture medium and centrifuge at 400 G for 5 minutes.
6. Discard the supernatant to remove residual cells.
7. Resuspend the sperm pellet in 0.5 mL of culture medium to obtain the sperm sample.

**S3** **The LabCASA workflow**

**Fig. S1** provides a clear description of the LabCASA software's processing workflow. The processing steps consist of nine distinct parts:

1. Real-time image acquisition: After activating the sperm motility analysis button, start to record images in real-time from the video stream.
2. Convert image type: Convert image format into 8-bit.
3. Image adjustments: Set image brightness at 50, contrast at 50, and gamma value at 0.45.
4. Threshold segmentation: Using the IMAQ AutoBThreshold module, a binary image is obtained through the clustering threshold segmentation algorithm.
5. Particle filter: Using the IMAQ ParticleFilter module, small particle impurities are filtered out through area detection to reduce interference in sperm analysis.
6. Particle analysis: Detecting the number of sperm in the image is accomplished through the IMAQ Particle Analysis Report.
7. Track objects: Using the Tracking Object module, the sperm detected in the previous step are tracked to obtain the motion coordinates of each sperm.
8. Calculate: Calculate the coordinates of each sperm to obtain parameters such as VCL, VAP, VSL, LIN, STR, and WOB.
9. Display data: Display the calculated data and display relevant parameters on the page.


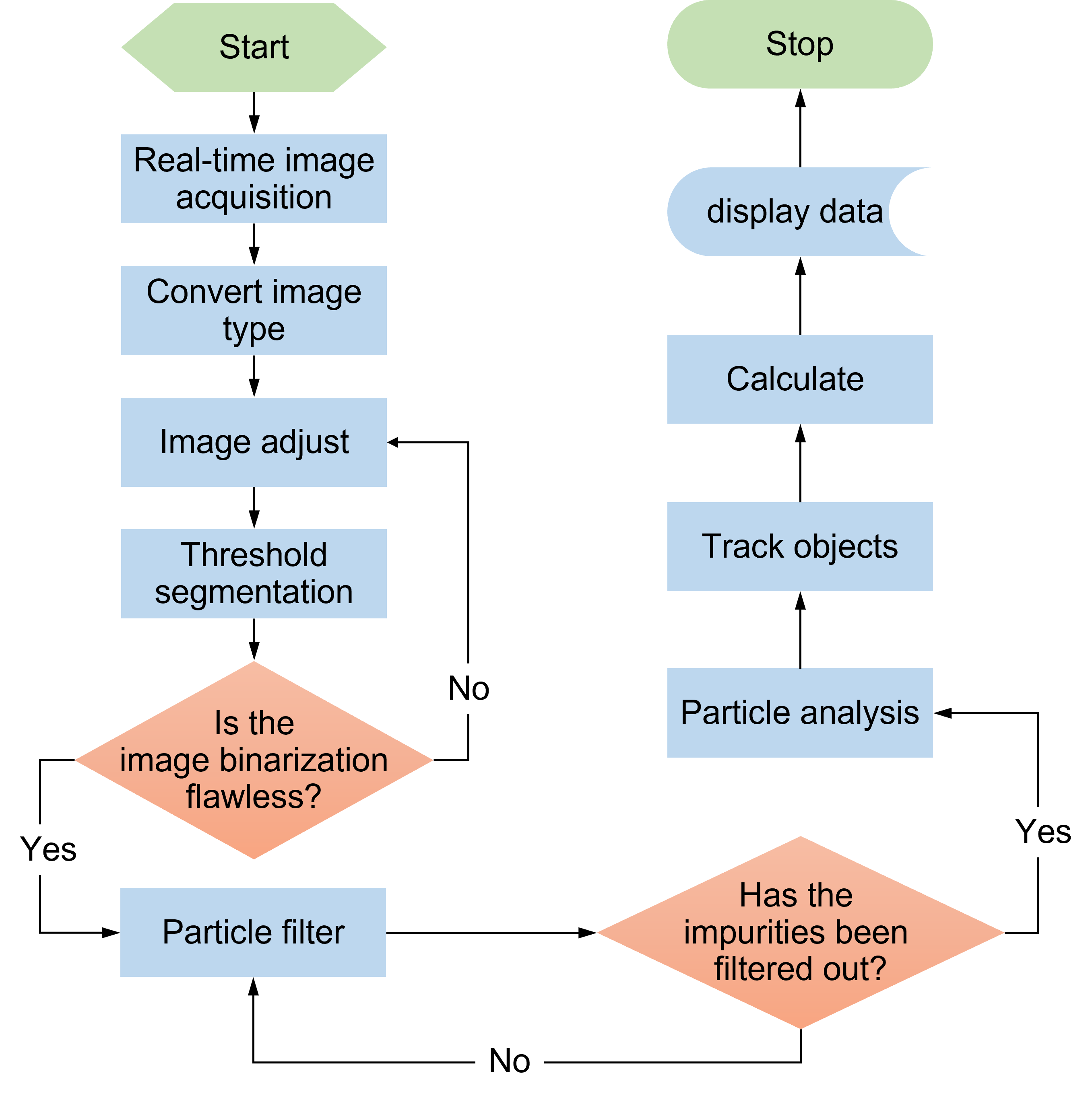


**Fig. S1** The LabCASA workflow.

**S4 Sampling frame rate and sperm motion parameters**

As shown in **Fig. S2** (a)-(c) respectively display the sperm motion states and trajectories at 10, 50, and 100 fps. With increasing frame rates, the reconstruction of sperm trajectories becomes more detailed. Therefore, VCL and VAP gradually increased. However, the position of the sperm in the first and last frames remained unchanged (frame extraction might cause the position of the last frame to differ), indicating that VSL would remain mostly unaffected. **Fig. S2** (d)-(i) illustrates the variation in sperm motion parameters at different frame rates. The data result shows that VCL and VAP gradually increase while VSL remains almost constant. According to the calculation formulas of LIN and STR, LIN and STR decrease gradually with increasing frame rate when VCL and VAP increase while VSL remains constant. However, the variation in WOB is not significant with changes in frame rate.


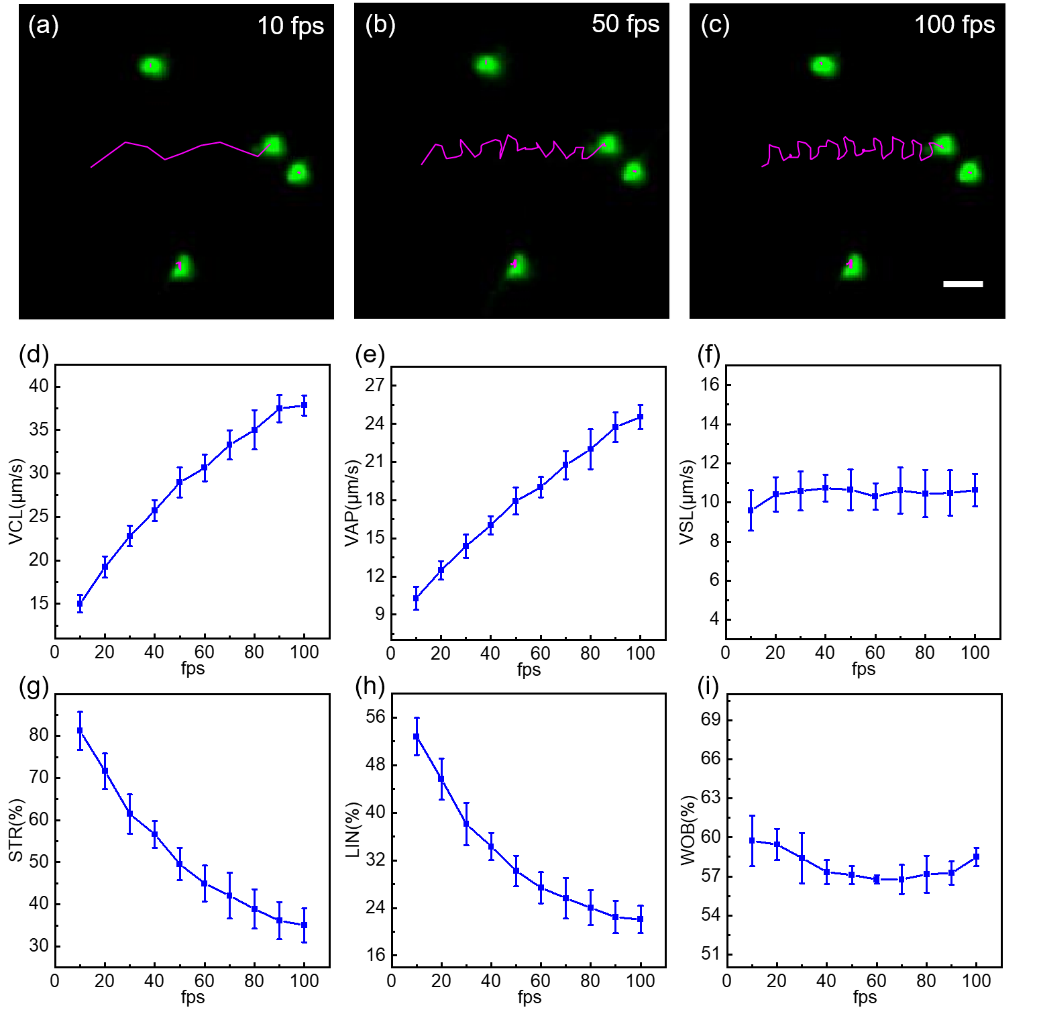


**Fig. S2** Impact of fps on assessing sperm motility parameters. (a)-(c) depict sperm trajectories at fps of 10, 50, and 100 respectively. (d)-(i) illustrate the variation in detected sperm motility parameters across different fps. Scale bar: 10 μm.

The performance of the computer is the main factor affecting the processing speed of LabCASA. It limits the video capture frame rate. **Fig. S3** illustrates the relationship between the set frame rate and the actual acquisition frame rate when running the LabCASA program on two computers. Under the same operating system and memory environment, a computer equipped with an i7-12700 CPU achieves real-time processing of approximately 32 fps. In contrast, a computer with an i7-10700 CPU can only achieve approximately 22 fps in real-time processing.


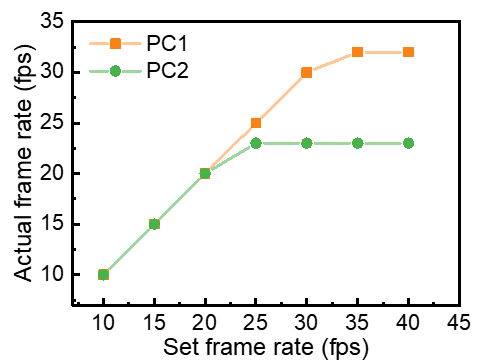


**Fig S3** The computer configuration impacts the frame rate of video processing in LabCASA.

**S4 The operation steps of TrackMate**

The operation steps of TrackMate are as follows:

1. Data Conversion: Video data needs to be converted into a compatible format for TrackMate, which may involve converting images to 8-bit, 16-bit, or 32-bit.
2. Filter Selection: Within TrackMate, users must manually select an appropriate filter for image processing. Commonly used filters include the LoG (Laplacian of Gaussian) filter.
3. Filter Parameter Adjustment: Parameters such as the estimated object diameter and quality threshold need to be adjusted manually to optimize the detection of sperm cells.
4. Tracker Selection: Within TrackMate, users must manually select an appropriate tracker after filter parameter adjustment. A commonly used tracker is the Simple LAP tracker.
5. Tracker Parameter Adjustment: Optimize tracking parameters such as "Linking max distance" set to 5 μm, "Gap-closing max distance" set to 5 μm, and "Gap-closing max frame gap" set to 2 for better trajectory results.
6. Further Programming: Additional programming may be required to process parameters such as VAP, which are not directly provided by TrackMate.

Throughout this process, clicking the "Next" button 10 times is required to obtain the processed data. These additional steps can add complexity and time to the operation of TrackMate, especially for researchers without programming experience.

In contrast, LabCASA simplifies the process by providing a more user-friendly interface and automating many of these steps. It eliminates the need for manual parameter adjustments by defaulting to preset values for brightness, contrast, and gamma, and it automatically tracks sperm motion while providing average motion parameters within one field of view.

**S5 Comparison of LAOM and MSM imaging.**

The illumination of the commercial microscope was modified with the same encoded LED array. The LED array-based Olympus microscope (LAOM) can achieve four kinds of imaging contrast. By changing the LED illumination patterns, it can achieve BF, DF, a combination of BF and DF, and Rheinberg imaging. **Fig. S4** compares the imaging effects of MLM and LAOM. **Fig. S4(a1)-(d1)** shows MLM under BF, DF, combined BF and DF, and Rheinberg conditions. **Fig. S4 (a2)-(d2)** show LAOM under the same conditions. Comparing the four kinds of imaging modes of LAOM, sperm imaging quality is very close.

Notably, due to diffraction effects, sperm in MSM are easier to identify. Under Rheinberg imaging, MSM outperforms LAOM in image quality. This is because MSM uses non-achromatic imaging lenses, which cause color fringing. This enhances the contrast of sperm details. These different imaging modes demonstrate consistency with our system, validating the performance and effectiveness of our approach.


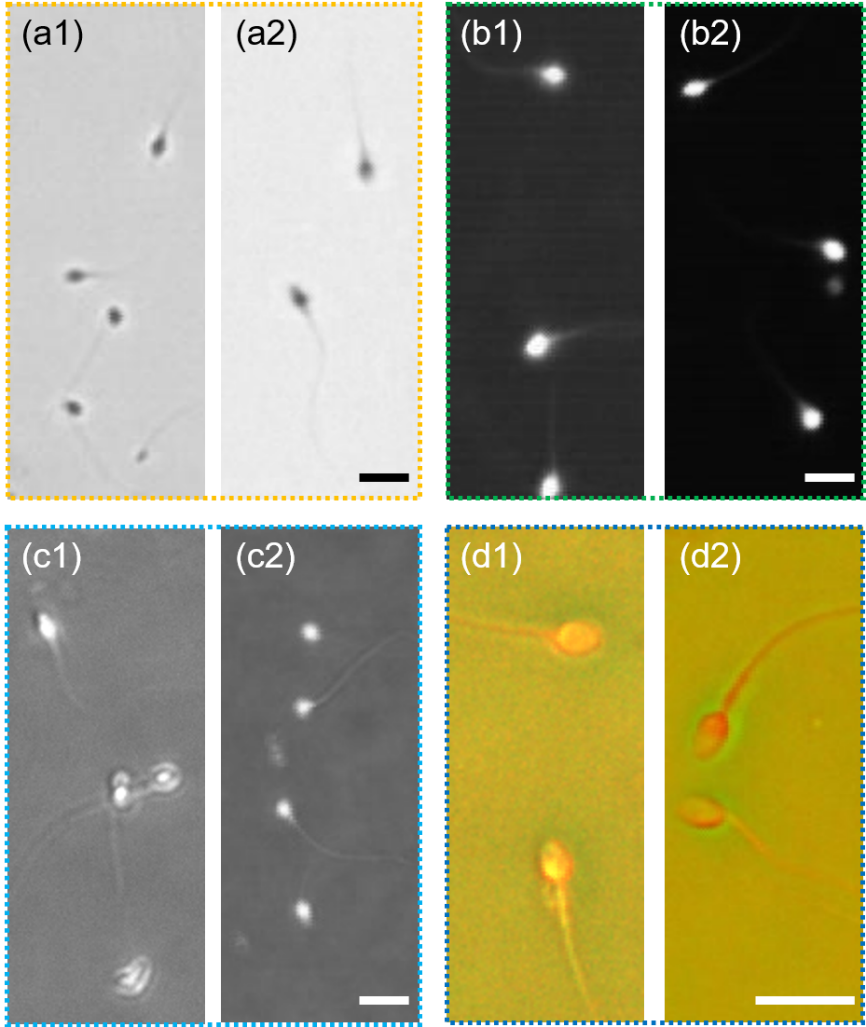


**Fig. S4** Multi-contrast imaging of sperm using MSM and LAOM. (a1)-(c1) Images were taken under a 10X objective in BF, DF, and a combination of BF and DF using MSM. (d1) Rheinberg imaging under a 20X objective using MSM. (a2)-(c2) Images were taken under a 10X objective in BF, DF, and a combination of BF and DF using LAOM. (d2) Rheinberg imaging under a 20X objective using LAOM. Scale bar: 10 μm.
